# *In Vivo* Biomarker Analysis of the Effects of Intranasally Dosed PC945, a Novel Antifungal Triazole, on Aspergillus fumigatus Infection in Immunocompromised Mice

**DOI:** 10.1128/AAC.00124-17

**Published:** 2017-08-24

**Authors:** Genki Kimura, Takahiro Nakaoki, Thomas Colley, Garth Rapeport, Pete Strong, Kazuhiro Ito, Yasuo Kizawa

**Affiliations:** a Laboratory of Physiology and Anatomy, Nihon University School of Pharmacy, Funabashi, Japan; b Pulmocide Ltd., London, United Kingdom

**Keywords:** Aspergillus fumigatus, azole, intranasal, galactomannan, IL-17, malondialdehyde, triazole

## Abstract

PC945 is a novel triazole optimized for lung delivery, and the objective of this study is to determine the effects of intranasally dosed PC945 on Aspergillus fumigatus infection and associated biomarkers in immunocompromised mice. PC945, posaconazole, or voriconazole was administered intranasally once daily on days 0 to 3 (early intervention) or days 1 to 3 (late intervention) postinfection in temporarily neutropenic A/J mice infected intranasally with A. fumigatus, and bronchoalveolar lavage fluid (BALF) and serum were collected on day 3. The effects of extended prophylaxis treatment (daily from days −7 to +3 or days −7 to 0) were also compared with those of the shorter treatment regimens (days −1 to +3 or days −1 and 0). Early and late interventions with PC945 (2.8 to 350 μg/mouse; approximately 0.11 to ∼14 mg/kg of body weight) were found to inhibit lung fungal loads and to decrease the concentrations of galactomannan (GM) in both BALF and serum as well as several biomarkers in BALF (interferon gamma [IFN-γ], interleukin-17 [IL-17], and malondialdehyde) and serum (tumor necrosis factor alpha [TNF-α] and IL-6) in a dose-dependent manner and were >3- and >47-fold more potent than intranasally dosed posaconazole and voriconazole, respectively. Furthermore, extended prophylaxis with low-dose PC945 (0.56 μg/mouse; 0.022 mg/kg) was found to inhibit fungal loads and to decrease the concentrations biomarkers more potently than did the shorter treatment regimens. Thus, PC945 dosed intranasally once daily showed potent antifungal effects, and the effects of PC945 accumulated upon repeat dosing and were persistent. Therefore, PC945 has the potential to be a novel inhaled therapy for the treatment of A. fumigatus infection in humans.

## INTRODUCTION

The incidence of fungal infections has increased substantially over the past 2 decades, and invasive forms are leading causes of morbidity and mortality, especially among immunocompromised or immunosuppressed patients. Alternatively, chronic lung infections with Aspergillus, such as previous infection with tuberculosis (1) or pulmonary inflammatory diseases, can leave patients with poor lung function and extensive and permanent lung structural changes (2–4).

Systemic triazole therapy is the basis for treatment of infections with Aspergillus, but the adverse effects of itraconazole, voriconazole, and posaconazole are well characterized and thought to be a consequence of the pharmacological effects of the compounds on host tissues (5, 6). Furthermore, notable drug interactions for voriconazole due to the inhibition of hepatic P450 enzymes make clinical use challenging, and indeed, the variability in the exposure of the triazoles via the oral route necessitates the need for close therapeutic drug monitoring and limits the use of triazole therapy prophylactically in at-risk groups (7, 8). In addition, Aspergillus colonization following infection occurs in a preexisting lung cavity due to tuberculosis or on airway surfaces where the fungus first is deposited and grows, and it is difficult to deliver and maintain the appropriate level of the drug after oral treatment (9). Furthermore, mutation induction by antimicrobial agents is an emerging problem worldwide; however, high-level and continuous exposure to antimicrobial agents is known to decrease the risk of mutation induction (10), although systemic treatment hardly achieves a high level of exposure in the lung cavity continuously. Thus, there are several advantages of topical treatment over oral/systemic treatment that alter the risk/benefit ratio of treatment favorably.

Amphotericin B is the only antifungal agent administered via the inhaled route (11, 12) because this agent has poor oral absorption, and its use as an intravenous infusion is complicated by adverse effects on the kidney (13). Inhaled amphotericin is well tolerated in lung transplant patients and does not interact with immunosuppressive drugs, although it has been reported to cause bronchospasm in severe asthmatics (14). Recently, nebulized voriconazole has been reported to produce improved therapeutic outcomes in patients after the failure or withdrawal of conventional treatment regimens due to adverse effects (15). Furthermore, inhaled voriconazole has also been tested on severe pulmonary infection with Scedosporium apiospermum in an adolescent with cystic fibrosis and confirmed to be safe and effective (16). Recently, a dry-power formulation of voriconazole for the treatment of respiratory fungal infection was developed (17). However, both voriconazole and amphotericin have very short durations of action in the lungs (short lung residence times) and require high doses and frequent administration.

An optimized compound for tropical delivery should have prolonged lung tissue residence with limited systemic exposure to display a better adverse-effect profile and eradicate invasive aspergillosis due to high-concentration exposure. Itraconazole is another triazole used to treat Aspergillus infection. McConville et al. demonstrated previously that single-dose nebulized itraconazole maintained high concentrations in the lung in mice for more than 24 h (18), and this was also demonstrated in rats (19). Furthermore, the survival rates of the group of mice receiving nebulized itraconazole were significantly higher than those of the group of mice receiving oral itraconazole administration (20). Since those reports, new formulations of itraconazole for topical treatment, such as nanoparticles (21, 22), polymeric micelles (23), itraconazole-loaded nanostructured lipid carriers (24), and dry powder (25), have been developed, although the *in vivo* antifungal activities of these inhaled products have not been reported formally. It is worth noting that inhaled PUR1900 (iSPERSE-formulated itraconazole; Pulmatrix), being developed for fungal infection in patients with cystic fibrosis, was recently reported to demonstrate higher lung exposure and lower systemic exposure than oral dosing in rats (26).

**FIG 1:**
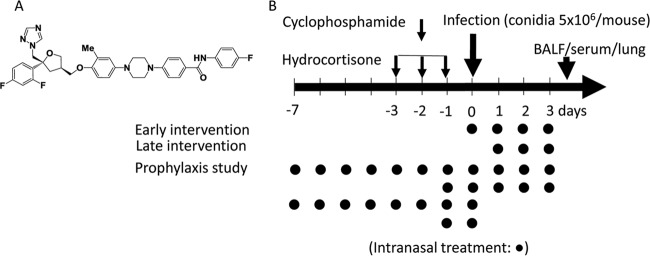
Chemical stricture of PC945 (A) and experimental protocol with the dosing regimen (B). A. fumigatus (ATCC 13073) and compounds (aqueous suspension) were given intranasally.

4-[4-(4-{[(3*R*,5*R*)-5-(2,4-Difluorophenyl)-5-(1H-1,2,4-triazol-1-ylmethyl)oxolan-3-yl]methoxy}-3-methylphenyl)piperazin-1-yl]-*N*-(4-fluorophenyl)benzamide (PC945) is a novel antifungal triazole (27, 28) (Fig. 1A), which has been shown to have potent antifungal activity against itraconazole-susceptible and -resistant Aspergillus fumigatus isolates with inhibition of the enzyme lanosterol 14α-demethylase (CYP51A1). PC945 has been designed to deliver high local concentrations; retention in cells, offering a long duration of action; minimal systemic exposure with poor oral availability; and high protein plasma binding. Thus, the aim of this study is to compare the antifungal effects of intranasal (i.n.) PC945 with those of intranasally dosed voriconazole and posaconazole quantitatively by using several biomarkers such as galactomannan (GM) and cytokines in temporarily neutropenic mice infected with A. fumigatus.

## RESULTS

### MICs for strain ATCC 13073.

The susceptibility of A. fumigatus (ATCC 13073) to PC945 was determined according to EUCAST and CLSI methods and compared with those to voriconazole and posaconazole. In the EUCAST test, MIC values of PC945, posaconazole, and voriconazole were 0.094, 0.094, and 0.50 mg/liter, respectively. According to the CLSI test, MIC values of PC945, posaconazole, and voriconazole were 0.031, 0.031, and 0.50 mg/liter, respectively. Thus, in both tests, the ATCC 13073 strain was highly susceptible to PC945, which was equal to posaconazole and 5.3- and 16-fold more potent than voriconazole according to EUCAST and CLSI tests, respectively.

### Effects of intranasally dosed PC945 in early and late interventions on fungal loads and biomarkers of A. fumigatus-infected mice.

**FIG 2:**
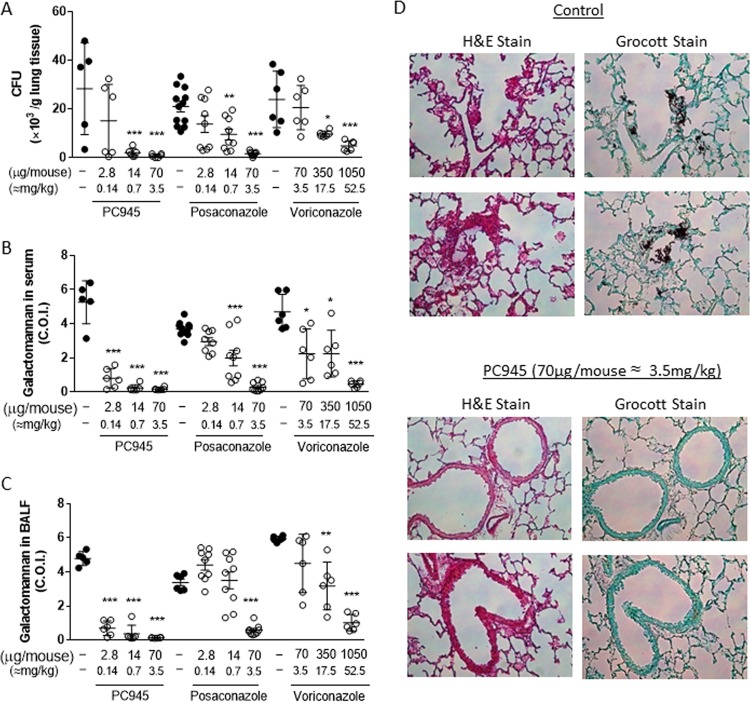
Effects of intranasally dosed PC945, posaconazole, or voriconazole (early intervention) on biomarkers in A. fumigatus-infected mice. Shown are fungal burdens in lung tissue (A), galactomannan levels in serum (B), galactomannan levels in BALF (C), and hematoxylin-eosin (H&E) staining and Grocott fungus staining (magnification, ×200) of lung sections (D). Compounds were dosed on day 0 (30 min before infection) and on days 1, 2, and 3 postinfection, and lungs, BALF, and serum samples were collected 6 h after the final dose. *, *P* < 0.05; **, *P* < 0.01; ***, *P* < 0.001 by a Kruskal-Wallis test (Dunn's comparison as *post hoc* analysis).

**FIG 3:**
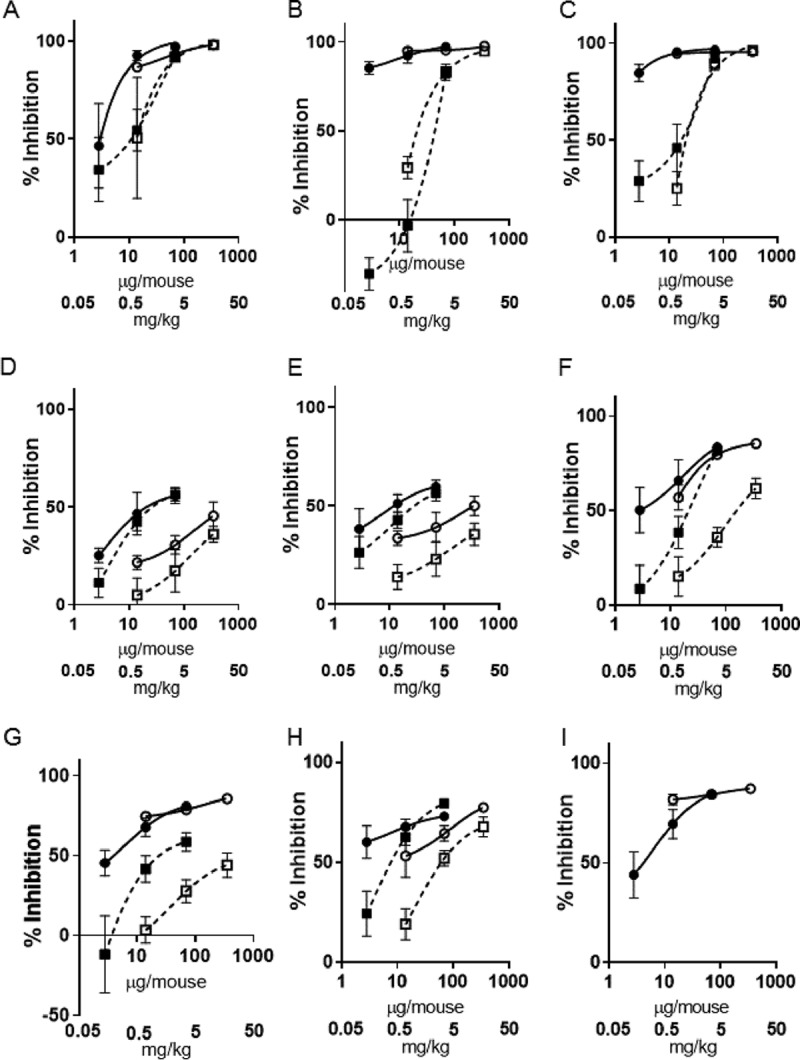
Inhibitory effects of intranasally dosed PC945 (closed circles, early intervention; open circles, late intervention) and posaconazole (closed squares, early intervention; open squares, late intervention) on biomarkers in A. fumigatus-infected mice. Shown are fungal burdens in lung tissue (A), galactomannan levels in serum (B), galactomannan levels in BALF (C), neutrophils in BALF (D), macrophages in BALF (E), IL-17 levels in BALF (F), MDA levels in BALF (G), IFN-γ levels in BALF (H), and IL-6 levels in serum (I).

A suspension of PC945 in isotonic saline (2.8, 14, 70, or 350 μg/mouse; equivalent to 0.11, 0.56, 2.8, or 14 mg/kg of body weight, respectively) was dosed by intranasal injection once daily on days 0, 1, 2, and 3 postinfection. This “early-intervention” regimen was found to strongly inhibit fungal loads in the lung (CFU), with a 50% infective dose (ID_50_) value of 2.66 μg/mouse (0.13 mg/kg) (Fig. 2A and 3A and Table 1; see also Table S1 in the supplemental material), and also to decrease GM concentrations in both bronchoalveolar lavage fluid (BALF) and serum in a dose-dependent manner (ID_50_s of <2.8 μg/mouse [<0.14 mg/kg] for both), as 85% inhibition was observed at 2.8 μg/mouse (Fig. 2B and C and 3B and C, Table 1, and Table S1). Notably, 70 μg/mouse (3.5 mg/kg) of PC945 produced an almost complete inhibition of fungal loads in the lung and GM in BALF and serum (Fig. 2B and C), which was confirmed by histology using Grocott fungus staining (Fig. 2D). PC945 also inhibited inflammatory cell accumulation in BALF; gamma interferon (IFN-γ), interleukin-17 (IL-17), and malondialdehyde (MDA) (an oxidative stress marker) levels in BALF; and tumor necrosis factor alpha (TNF-α) and IL-6 levels in serum, all of which were elevated by A. fumigatus infection (Fig. 3D to I, Table 1, and Table S2). Intranasal dosing with either posaconazole or voriconazole also inhibited infection, but neither compound was as potent as PC945 (Fig. 2A to C and 3A to H, Table 2, and Table S3). For example, PC945 was more than 109 and 19 times more potent than voriconazole and posaconazole, respectively, against GM in BALF and 105 and 3 times more potent than voriconazole and posaconazole against CFU in lung tissue (Table 1).

**TABLE 1:** ID_50_ values for early intervention and late intervention with PC945, voriconazole, or posaconazole on biomarkers in BALF and serum in Aspergillus fumigatus-infected, immunocompromised, neutropenic mice^*a*^

Biomarker	ID_50 intranasal_ (μg/mouse) (ID_50 intranasal_ [mg/kg] [95% confidence interval {μg/mouse}])				
	Early intervention			Late intervention	
	PC945	Voriconazole	Posaconazole	PC945	Posaconazole
CFU in lung	2.66 (0.13 [1.09–6.46])	278 (13.9 [160–485])	8.23 (0.41 [3.98–17.0])	<14 (0.70 [NA])	12.0 (0.60 [3.34–42.8])
GM in BALF	<2.8 (0.14 [NA])	304 (15.2 [176–525])	50.6 (2.53 [19.6–130])	<14 (0.70 [NA])	24.2 (1.21 [17.1–34.2])
GM in serum	<2.8 (0.14 [NA])	132 (6.58 [61.0–283])	11.5 (0.58 [6.25–21.3])	<14 (0.70 [NA])	23.6 (1.18 [14.7–37.8])
Neutrophils in BALF	23.6 (1.2 [12.9–43.4])	451 (22.5 [231–880])	31.5 (1.57 [20.9–47.5])	281 (14.1 [166–476])	549 (27.4 [284–1,060])
Macrophages in BALF	14.0 (0.70 [7.21–27.3])	918 (45.9 [492–1,690])	26.1 (1.30 [16.2–42.1])	168 (8.41 [88.1–321])	491 (24.6 [268–901])
IL-17 in BALF	4.2 (0.21 [2.11–8.37])	943 (47.1 [352–2,530])	20.6 (1.03 [11.8–36.0])	12.5 (0.62 [8.63–18.0])	154 (7.72 [93.3–256])
IFN-γ in BALF	3.04 (0.15 [1.62–5.71])	585 (29.3 [266–1,290])	9.43 (0.47 [6.01–14.8])	20.9 (1.04 [11.4–38.1])	82.0 (4.10 [52.9–127])
MDA in BALF	4.83 (0.24 [3.04–7.69])	669 (33.4 [416–1,076])	36.8 (1.84 [13.3–101])	6.29 (0.31 [3.95–10])	348 (17.4 [195–619])
TNF-α in serum	3.33 (0.17 [1.90–5.84])	ND (ND [NA])	ND (ND [NA])	6.04 (0.30 [3.55–10.3])	ND (ND [NA])
IL-6 in serum	4.69 (0.23 [2.68–8.21])	ND (ND [NA])	ND (ND [NA])	3.94 (0.20 [2.37–6.56])	ND (ND [NA])

a ND, not done; NA, not applicable; GM, galactomannan; BALF, bronchoalveolar lavage fluid; IFN-γ, interferon gamma; IL-17, interleukin 17; MDA, malondialdehyde.

**TABLE 2:** Effects of repeated prophylactic dosing of PC945 on fungal loads (CFU) in lung and galactomannan concentrations in BALF and serum in Aspergillus fumigatus-infected, immunocompromised, neutropenic mice^*a*^

Treatment regimen	Dose (μg/mouse)	Mean value (% inhibition of response) ± SE		
		CFU (mg of lung)	GM in BALF (COI)	GM in serum (COI)
Vehicle + conidia	None	34.7 ± 10.7	5.1 ± 0.9	4.3 ± 1.0
Daily administration of drug on days:				
−7 to +3	0.11	8.3 ± 2.0 (76)	2.6 ± 0.36 (49)	1.8 ± 0.43 (58)
−1 to +3	0.11	9.5 ± 3.3 (73)	2.8 ± 0.71 (45)	2.2 ± 0.69 (49)
−7 to +3	0.56	5.0 ± 2.3 (86)	1.7 ± 0.39 (67)	1.4 ± 0.20 (67)
−1 to +3	0.56	6.7 ± 1.7 (81)	2.3 ± 0.52 (55)	1.7 ± 0.59 (60)
−7 and 0	0.56	6.1 ± 2.8 (82)	2.2 ± 0.61 (57)	1.6 ± 0.41 (63)
−1 and 0	0.56	13.1 ± 2.6 (62)	4.5 ± 0.50 (12)	4.0 ± 0.88 (7)

a The data for fungal load and GM are shown as means ± standard errors (percent inhibition with respect to the vehicle) (*n* = 5). COI, cutoff index.

Using the “late-intervention” regimen (once daily on days 1, 2, and 3 postinfection), PC945 (14, 70, or 350 μg/mouse [0.70, 3.5, or 17.5 mg/kg], respectively) also dose-dependently inhibited fungal loads in lung tissue (CFU) and decreased GM concentrations in both BALF and serum in a dose-dependent manner (Fig. 3A to C and Table S1). At the lowest dose evaluated (14 μg/mouse [0.70 mg/kg]), PC945 showed more than 87% inhibition of the tissue fungal load (CFU) and GM, and therefore, ID_50_ values were not calculable, and the inhibitory effects were similar to those of the early intervention (Table 1). PC945 in this regimen also inhibited inflammatory cell accumulation in BALF; the levels of IFN-γ, IL-17, and MDA in BALF; and the levels of TNF-α and IL-6 in serum (Fig. 3D to I and Table S2). The same late-intervention dosing regimen with posaconazole also inhibited infection in a dose-dependent manner, but it was not as potent as PC945. For example, the effect of PC945 on IL-17, IFN-γ, and MDA was 12-, 4-, and 55-fold more potent, respectively, than that of posaconazole based on the ID_50_ values (Table 1).

### Effects of extended-prophylaxis dosing of PC945 on biomarkers of A. fumigatus-infected mice.

**FIG 4:**
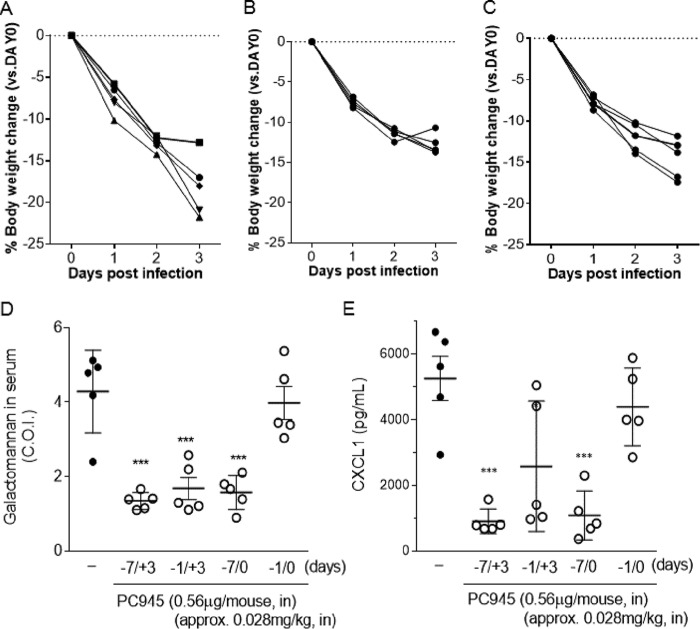
Effects of extended prophylaxis of intranasal PC945. (A to C) Body weight loss postinfection in control infected mice (A), mice treated with PC945 on days −7 to +3 (B), and mice treated with PC945 on days −1 to +3 (C). (D and E) Galactomannan levels in serum (D) and CXCL1 levels in BALF (E) from mice treated with PC945 on days −7 to +3 (−7/+3), days −1 to +3 (−1/+3), days −7 to 0 (−7/0), and days −1 and 0 (−1/0). *, *P* < 0.05; ***, *P* < 0.001 (versus the infection control, as determined by a Kruskal-Wallis test [Dunn's comparison as *post hoc* analysis]).

Body weight was reduced by approximately 17.5% during 3 days postinfection in the control group. Treatment from days −1 to +3 improved body weight loss only slightly, but extended prophylaxis (days −7 to +3) showed remarkable improvement (Fig. 4A to C). In addition, extended prophylaxis with PC945 (days −7 to +3) was found to inhibit fungal loads in lung tissue (CFU), GM concentrations in both BALF and serum, and cytokine concentrations slightly more than the shorter-period treatment (days −1 to +3). Furthermore, extended prophylaxis with PC945 (days −7 to +3) achieved greater inhibitory effects on fungal loads and biomarkers at much lower doses (0.56 and 0.11 μg/mouse [0.028 mg/kg and 0.0056 mg/kg], respectively) than those used in previous early/late-intervention studies (compare Tables 2 and 3 to Tables S1 and S2 in the supplemental material, and compare Fig. 3D to 2B). For example, PC945 showed 46% inhibition of CFU in lung at 2.8 μg/mouse (0.14 mg/kg) with the early intervention (days 0 to +3) (Table S1), and the same level of inhibition was observed at 0.28 μg/mouse (0.014 mg/kg) with extended prophylaxis treatment (days −7 to +3) (Table 2), suggesting that extended prophylaxis was 10-fold more potent than early intervention. This was also confirmed for serum TNF-α and IL-6, where extended prophylaxis (days −7 to +3) was 7- and 14-fold more potent, respectively, than early intervention, as determined by ID_50_ values.

**TABLE 3:** Effects of repeated prophylactic dosing of PC945 on macrophage and neutrophil accumulation in BALF of Aspergillus fumigatus-infected, immunocompromised, neutropenic mice^*a*^

Treatment	Dose (μg/mouse)	Mean no. of cells (10^5^) in BALF/ml ± SE (% inhibition)		Mean concn of biomarker in BALF ± SE (% inhibition)			Mean concn of biomarker (pg/ml) in serum ± SE (% inhibition)	
		Macrophages	Neutrophils	CXCL1 (ng/ml)	IL-17 (pg/ml)	MDA (μg/ml)	IL-6	TNF-α
Vehicle + conidia	None	0.60 ± 0.082	0.48 ± 0.14	5.3 ± 1.4	21.9 ± 10.4	1.3 ± 0.23	247 ± 80.1	32.5 ± 9.59
Daily administration of drug on days:								
−7 to +3	0.11	0.50 ± 0.062 (17)	0.41 ± 0.15 (15)	2.3 ± 0.48 (57)	11.4 ± 2.2 (48)	0.93 ± 0.14 (28)	161 ± 25.2 (35)	20.0 ± 3.02 (38)
−1 to +3	0.11	0.53 ± 0.058 (12)	0.40 ± 0.025 (17)	3.2 ± 1.4 (40)	14.6 ± 4.3 (33)	1.0 ± 0.14 (23)	172 ± 21.5 (30)	19.9 ± 6.44 (61)
−7 to +3	0.56	0.46 ± 0.070 (23)	0.36 ± 0.054 (25)	0.90 ± 0.34 (83)	8.2 ± 1.6 (63)	0.66 ± 0.16 (49)	101 ± 18.1 (59)	13.0 ± 2.82 (60)
−1 to +3	0.56	0.49 ± 0.062 (18)	0.38 ± 0.082 (21)	2.6 ± 1.8 (51)	12.7 ± 4.4 (42)	0.78 ± 0.20 (40)	143 ± 53.2 (42)	17.4 ± 10.2 (46)
−7 and 0	0.56	0.49 ± 0.072 (18)	0.38 ± 0.046 (21)	1.1 ± 0.67 (79)	9.9 ± 1.8 (55)	0.71 ± 0.15 (45)	121 ± 28.1 (51)	15.0 ± 5.20 (54)
−1 and 0	0.56	0.54 ± 0.10 (10)	0.43 ± 0.11 (10)	4.4 ± 1.1 (17)	19.6 ± 3.2 (11)	1.2 ± 0.13 (14)	215 ± 27.9 (13)	27.3 ± 4.64 (16)

a Data are shown as means ± standard errors (percent inhibition with respect to the vehicle) (*n* = 5). CXCL1, chemokine (C-X-C motif) ligand 1.

A more marked difference between extended prophylaxis and shorter treatment was observed between days −7 to 0 and days −1 to 0 (Fig. 4D and E and Tables 2 and 3). For example, percentages of inhibition with 0.56 μg/mouse were 63% for days −7 to 0 and 7% for days −1 to 0 for serum GM, 57% for days −7 to 0 and 12% days −1 to 0 for BALF GM, and 79% for days −7 to 0 and 17% for days −1 to 0 for BALF CXCL1 (Tables 2 and 3).

## DISCUSSION

PC945 is a novel antifungal triazole that has been found to be an inhibitor of planktonic growth and bronchial epithelial cell infection by itraconazole-susceptible and -resistant A. fumigatus strains *in vitro*, with persistent action (27). In this study, we demonstrated that topical once-daily treatment with PC945 was highly effective against the fungal burden in lung tissue and several biomarkers (galactomannan and cytokines) in serum and BALF in temporarily neutropenic mice infected with A. fumigatus compared with intranasally administered posaconazole and voriconazole.

Existing triazole-type antifungal medicines, which are predominantly dosed either orally or systemically, have several common problems, such as systemic unwanted effects and poor and variable drug concentrations achieved at the site of infection (7, 29–31). Therefore, targeted pulmonary delivery by aerosolization of antifungals has gained attention to avoid systemic side effects and to achieve high local concentrations at the primary site of infection. Tolman and colleagues demonstrated that prophylaxis with an aerosolized aqueous intravenous formulation of voriconazole significantly improved survival and limited the extent of invasive disease, as assessed by histopathology, in an invasive pulmonary murine model (32). Interestingly, mice that received aerosolized voriconazole had a survival advantage over controls and those treated with amphotericin B.

We also demonstrated that intranasally dosed voriconazole provided dose-dependent inhibition of GM in serum and BALF and lung tissue fungal burdens in A. fumigatus-infected temporarily neutropenic mice (Fig. 2A; see also Table S1 in the supplemental material). However, voriconazole required a very high dose (1,050 μg/mouse; 52.5 mg/kg) to inhibit these measures by >80%, and the effects of voriconazole on CFU in lung, GM in BALF, and GM in serum were 105-, >109-, and >47-fold weaker, respectively, than those of PC945 (Table 1). In addition, PC945 was 3-, >18-, and >4-fold more potent than posaconazole, which was reported to have longer tissue retention than voriconazole (33–37), against CFU in lung, GM in BALF, and GM in serum (Table 1 and Fig. 2), despite the fact that posaconazole and PC945 showed similar MICs for this strain in broth microdilution assays.

The anti-Aspergillus activity of PC945 *in vivo* was investigated in mice by using two treatment regimes. PC945 was administered to animals on either days 0, 1, 2, and 3 postinfection (early intervention) or days 1, 2, and 3 postinfection (late intervention). PC945 showed marked effects on fungal burdens in lung and biomarkers in serum and BALF by both dosing regimens, and there was no significant difference in efficacies between the two dosing regimens. Especially, mice treated with PC945 intranasally (early intervention) demonstrated no or few fungi with dark Grocott silver staining in the alveolar area by histology (Fig. 2D), which was strong evidence that intranasally administered PC945 efficiently eliminated the fungus in the lung. Thus, PC945 was able to inhibit local fungus growth quickly and consequently reduced systemic invasion by A. fumigatus.

In addition, we also investigated the effects of extended prophylaxis with PC945 on biomarkers in A. fumigatus-infected mice. PC945 was administered intranasally at 0.11 or 0.56 μg/mouse from either 7 days before infection to 3 days postinfection or days −7 to 0, and the effects were compared with those of the shorter treatment (days −1 to +3 or days −1 to 0, respectively). In these studies, the dose was almost 25-fold lower than that used in the early- and late-intervention studies discussed above. Prophylaxis with PC945 was found to inhibit fungal loads in the lung, GM concentrations in both BALF and serum, and cytokine concentrations, and the inhibitory effects of prophylaxis (days −7 to +3) were more than 10-fold more potent than those of either early or late intervention (Tables 2 and 3 and Tables S1 and S2). Furthermore, the data suggested an accumulation of antifungal effects in the lung upon repeat dosing, since 7 days of prophylaxis (days −7 to +3 or −7 to 0) produced greater antifungal effects than did single-day prophylaxis (days −1 to +3 or −1 to 0). In particular, the persistence of PC945 action in the lung, which could contribute to the accumulation of effects, was suggested by the observation that prophylaxis treatment (days −7 to 0) generated superior antifungal effects than those that resulted from treatment from days −1 to 0, as the antifungal effects were evaluated on day 3 after treatment was stopped.

In our temporarily neutropenic model, concentrations of IFN-γ and IL-17 in BALF and TNF-α and IL-6 in serum were significantly increased in BALF from Aspergillus-infected mice. Significant increases in IL-17, IL-8, and/or IFN-γ concentrations in response to Aspergillus antigen or infection have been observed in human epithelial cells or blood mononuclear cells (38–40). Studies in an *in vivo* murine model also showed that lung fungal growth was associated with elevated lung IL-17 and IFN-γ levels (41). Bronchial epithelial cells are known to be a major source of IL-8 and IL-17 *in vitro*. IL-8 (KC/CXCL1 in mice) is a chemotactic factor for the activator of neutrophils, which is stimulated with IL-17 in epithelial cells. In addition, IFN-γ is known to be an antimicrobial factor. Thus, these cytokines are important factors for innate immunity, although these cytokines are also acknowledged as biomarkers of Aspergillus infection, as their levels are elevated after infection/invasion. In this study, we demonstrated that PC945 strongly inhibited the A. fumigatus infection-induced elevation of IL-17, IFN-γ, and CXCL1 levels in mice with greater potency than either posaconazole or voriconazole. This does not mean that PC945 inhibited Aspergillus infection-induced innate immunity, and the data are believed to demonstrate that PC945 showed reductions in the levels of these cytokines as a consequence of the inhibition of fungus growth in lungs. In fact, we found that PC945 did not inhibit IL-17, IFN-γ, CXCL1, and other cytokines stimulated by nonfungus stimulation (Cytostim-T lymphocyte activator) in human peripheral blood mononuclear cells (PBMCs) (see Fig. S1 in the supplemental material).

In addition, we also found that the level of the oxidative stress marker MDA was increased in BALF from Aspergillus-infected mice. Increased formation of oxygen radicals was previously shown for alveolar macrophages (AMs) challenged with Cryptococcus neoformans, Candida albicans, and A. fumigatus, without an alteration of AM cell viability (42). In addition, human epithelial cells are a potential source of reactive oxygen in response to Dectin-1 (43). Interestingly, some Aspergillus fumigatus strains have detoxifying systems for reactive oxygen species (ROS), such as catalases and superoxide dismutase, which are essential for virulence (44). Furthermore, A. fumigatus is known to have the ability to generate ROS by itself (45), which may contribute to the invasiveness of A. fumigatus and the inflammatory response of host cells.

The limitation of this study was a lack of pharmacokinetic measurements of PC945 in mice during infection to explain the superiority of PC945 compared to voriconazole and posaconazole. PC945 has been optimized for topical treatment of the lung to maximize local exposure and minimize systemic exposure. Systemic concentrations of the drug are therefore not a useful surrogate marker to help explain the different antifungal efficacies of compounds. However, we have some data demonstrating that limited systemic exposure occurs. In preliminary studies using noninfected A/J mice, intranasal treatment with PC945 (70 μg/mouse [approximately 3.5 mg/kg] i.n.) showed limited but measurable systemic exposure (PC945 contents in serum of 0.64 ± 0.61 ng/ml at 12 h posttreatment [detectable in 7 out of 8 animals] and 0.26 ± 0.13 ng/ml at 24 h posttreatment [detectable in 4 out of 8 animals]). In addition, PC945 dosed intratracheally with 40 μl of a 2 mg/ml aqueous suspension showed plasma concentration ranges of 37.8 to 55.8 ng/ml at 2 h postdose and 2.1 to 2.8 ng/ml at 24 h postdose. Under the same conditions, the plasma concentrations achieved with posaconazole were 2.96 to 23.6 ng/ml at 2 h postdose and 15.6 to 125 ng/ml at 24 h postdose. Thus, after single doses, the maximum concentrations achieved are not too different, but the time profiles are hugely different with posaconazole, demonstrating a much higher area under the concentration-time curve (AUC) than is apparent for PC945. As PC945 has no or little oral availability (our unpublished data), exposure results from absorption through the respiratory tract (not by compounds accidentally swallowed). In addition, we carefully optimized the intranasal dosing volume, and based on data reported previously (46), approximately 60% of the administered dose will be deposited in the lung after intranasal treatment with 35 μl.

In addition, all compounds are water insoluble and administered topically (exposed to the respiratory tract directly). We therefore do not believe that water solubility is a key factor explaining the *in vivo* efficacies or topical exposure levels. As shown by all fungal and cytokine biomarker results, PC945 was superior to posaconazole and voriconazole even though MIC values for this A. fumigatus strain were not so different. Our speculation is that PC945 has the following properties. First, PC945 has persistent action, as PC945 has been demonstrated to have a longer duration of action in bronchial epithelial cells and hypha (27). This might have contributed to the superior antifungal activity of the current once-daily regimen. Second, our rat study demonstrated that PC945 accumulated in the lung after repeat daily dosing (our unpublished data). In addition, we have demonstrated here that 7-day prophylactic treatment (using very low doses) produced much greater anti-Aspergillus activity than did prophylactic treatment for 1 day and also that the effects of 7-day prophylactic treatment are maintained if treatment ceases when Aspergillus is inoculated on day 0 (Fig. 4D and E and Tables 2 and 3). This is powerful pharmacodynamic evidence that the effects of PC945 accumulate upon daily dosing in mice and are maintained when dosing is terminated. Third, Baistrocchi and colleagues recently reported that posaconazole accumulated in granulocyte-type cells and that it enhanced synergic antifungal effects (by exposure to cellular posaconazole during phagocytosis) (47). Considering to persistent action of PC945, granulocytes/macrophages containing PC945 contributed to the further enhancement of the antifungal agent.

Thus, intranasally dosed PC945 showed more potent inhibition of fungal loads, GM concentrations, and A. fumigatus-dependent inflammation than did intranasal treatment with either posaconazole or voriconazole. The data suggest that the antifungal effects of PC945 in the lung accumulated upon repeat dosing and that these effects are persistent. Thus, PC945 has the potential to be a novel inhaled therapy for the prevention or treatment of A. fumigatus lung infection in humans.

## MATERIALS AND METHODS

### Materials.

PC945 was synthetized in Sygnature Discovery Ltd. (Nottingham, UK), and posaconazole and voriconazole were sourced from Apichem Chemical Technology Co. Ltd. (Zhejiang, China) and Tokyo Chemical Industry UK Ltd. (Oxford, UK), respectively. For *in vitro* assays, all articles were dissolved in dimethyl sulfoxide (DMSO) at 2 mg/ml as a stock solution. For *in vivo* assays, solid materials were directly suspended in physiological saline at 10 mg/ml and diluted with physiological saline after sonication.

### Strain.

A. fumigatus (ATCC 13073 [NIH 5233]) was purchased from the ATCC (Manassas, VA, USA).

### MIC determinations.

Antifungal susceptibility testing was performed based on EUCAST methods (48) in-house or based on CLSI methods (CLSI document M38-A) (49) by Eurofins Panlabs Inc. Stock solutions of test agents (2 mg/ml) were prepared in neat DMSO and serially diluted with neat DMSO to reach 200-fold concentrations of the desired compounds. These solutions were then applied to fungus growth medium at a 1:200 dilution, allowing the final DMSO concentration to be constant across the plate at 0.5% (vol/vol). The MIC was read as the lowest drug concentration that yielded no visible growth by eye or prevented any discernible growth (100% inhibition) according to each guideline.

### Animal model and treatment.

The preparation of the animal model and the treatment regimen are shown in Fig. 1B. Conidia of A. fumigatus (ATCC 13073 [NIH 5233]) grown on plates of Malt agar (Nissui Pharmaceutical, Tokyo, Japan) were aseptically dislodged from the agar plates and suspended in 0.1% agar. A/J mice (males, 5 weeks old) were dosed with hydrocortisone (125 mg/kg subcutaneously [s.c.]) (catalog number H4881; Sigma) on days 3, 2, and 1 before infection and with cyclophosphamide (250 mg/kg intraperitoneally [i.p.]) (catalog number C0768; Sigma) 2 days before infection to induce immunosuppression and temporary neutropenia, as previously reported (50). Neutropenic conditions were observed up to day 3 after cyclophosphamide injection and gradually recovered (our unpublished data). On day 0, animals were infected with the conidium suspension (1.67 × 10^8^ conidia ml^−1^ of physiological saline [30 μl intranasally {15 μl each into each nostril}]). Body weights were monitored daily. Animals, when demonstrating >20% weight loss, showed rough hair coat, difficulty breathing, head tilt, lethargy, trembling, and lower body temperature and, in most cases, died in the next days or a day after from our historical data. However, if body weight loss was <20%, the animals ate less and became less active, but many of the animals recovered at later time points. Therefore, in this study, if body weight fell >20% compared to that on day 0, animals were sacrificed by using a high dose of pentobarbital.

All animal studies and class II pathogen use were approved by the Ethics Review Committee for Animal Experimentation of Nihon University and the Microbial Safety Management Committee of the Nihon University School of Pharmacy (E-H25-001).

### Treatment.

Either PC945, voriconazole, or posaconazole, which was suspended in physiological saline, was administered intranasally (35 μl/mouse; approximately 17.5 μl in each nostril) once daily, on day 0 postinfection (30 min before conidium infection); days 1, 2, and 3 postinfection (early intervention); or days 1, 2, and 3 postinfection (late therapeutic intervention), as shown in Fig. 1B. The volume administered intranasally has been reported to achieve almost 60% deposition into lungs (46). Animals were terminally anesthetized 6 h after the last dose of the drug was administered on day 3 (Fig. 1B). BALF was collected through cannulated tracheas using physiological saline (51), blood was then collected via cardiac puncture, and lung tissue was removed for homogenate preparation. To investigate extended prophylaxis, PC945 was administered intranasally once daily, on days −7 to 3 or days −7 to 0, and the effects were compared with those of treatment on days −1 to 3 or days −1 and 0. As the injection volume was fixed and the body weight changed every day, especially after infection, the accurate dose unit was micrograms per mouse. However, as the average body weight after immunosuppression and just before infection was 20 g, we also calculated the estimated dose as milligrams per kilogram. The conversion of dosing units is shown in Table 4.

**TABLE 4:** Dose unit conversion

Concn in stock solution for intranasal treatment (mg/ml)	Dose unit conversion	
	μg/mouse	mg/kg (estimated as body wt of 20 g)
0.016	0.56	0.028
0.08	2.8	0.14
0.4	14	0.70
2	70	3.5
10	350	17.5

### Biomarker analysis.

GM concentrations in serum and BALF were determined by using Platelia GM-EIA kits (Bio-Rad Laboratories, Redmond, WA, USA). The cutoff index (COI) was calculated by using the formula cutoff index = optical density (OD) in the sample/OD in the cutoff control provided as part of the kit. For semiquantitative tissue fungal load analysis, 100 mg of lung tissue was removed aseptically and homogenized in 0.2 ml of low-nutrient 0.1% sterile water–agar to limit the growth of the fungus in homogenates on ice until all samples were processed. Homogenates were prepared by using a minicordless CG-4A homogenizer (Funakoshi Ltd., Tokyo, Japan) under mild conditions with 2 repeated cycles of 10 s of homogenization and 2 min of resting on ice. Serially diluted lung homogenates with sterile physiological saline were plated onto Malt agar plates (50 μl/plate) and incubated at 24°C ± 1°C for 72 to 96 h. We used room temperature and a longer culture period to avoid any sporulation during analysis. Colonies of A. fumigatus on each plate were counted, and the fungal titer is presented here as CFU per gram of lung tissue. In preliminary analyses, we found a good correlation between CFU and A. fumigatus copy numbers by PCR analysis (*P* < 0.001) (our unpublished data). The numbers of alveolar macrophages and neutrophils in BALF were determined by fluorescence-activated cell sorter (FACS) analysis (Epics Altra II; Beckman Coulter Inc., Fullerton, CA, USA) using anti-mouse MOMA2-fluorescein isothiocyanate (FITC) (macrophage) or anti-mouse 7/4 (neutrophil), as previously reported (51). Measurement of IFN-γ, IL-17, and CXCL1 levels in BALF and TNF-α and IL-6 levels in serum was performed by using a Quantikine mouse enzyme-linked immunosorbent assay (ELISA) kit (R&D Systems Inc., Minneapolis, MN, USA). MDA analysis was also performed by using an OxiSelect TBARS (thiobarbituric acid reactive substances) assay kit (MDA quantitation; Cell Biolabs Inc., San Diego, CA, USA). For histology, whole lungs were fully inflated by intratracheal perfusion with 10% paraformaldehyde in phosphate-buffered saline (PBS). Lungs were then dissected and placed into a fresh paraformaldehyde solution. Routine histological techniques were used to paraffin embed this tissue, and 4-μm sections of whole lung were stained with hematoxylin and eosin and a Grocott stain kit (silver stain kit, catalog number HT100A; Sigma).

### Statistical analysis.

Results are expressed as means ± standard errors of the means (SEM). Multiple comparisons were performed by analysis of variance (ANOVA) followed by Dunnett's multiple-comparison test by using the PRISM 6 software program (GraphPad Software Inc., San Diego, CA, USA). Comparisons between 2 groups were performed by using an unpaired *t* test with Welch's correction or a Mann-Whitney test. The ID_50_ value was also calculated from dose-response curves by nonlinear regression analysis with three-parameter fitting using the PRISM 6 software program when more than 3 doses were tested. To facilitate modeling, we kept the lower and maximal effects constant (as 0 and 100%). The 95% confidence intervals were also calculated. For cases where only two doses were tested, the estimated ID_50_ value was calculated by linear regression for information. Statistical significance was defined as a *P* value of <0.05.

## Supplementary Material

Supplemental material
