# Supplementary material for: In Vivo Biomarker Analysis of the Effects of Intranasally Dosed PC945, a Novel Antifungal Triazole, on Aspergillus fumigatus Infection in Immunocompromised Mice
Source: Antimicrob Agents Chemother. 2017 Aug 24;61(9):e00124-17. doi: 10.1128/AAC.00124-17 (PMC5571324; doi:10.1128/AAC.00124-17)
Supplement: Supplemental material [file supp_61_9_e00124-17__index.html]

Supplemental material 

# *In Vivo* Biomarker Analysis of the Effects of Intranasally Dosed PC945, a Novel Antifungal Triazole, on Aspergillus fumigatus Infection in Immunocompromised Mice

## Supplemental material

- Supplemental file 1 -

  Fig. S1 and Tables S1 to S3

  PDF, 684K
