# Supplementary material for: In Vivo Biomarker Analysis of the Effects of Intranasally Dosed PC945, a Novel Antifungal Triazole, on Aspergillus fumigatus Infection in Immunocompromised Mice
Source: Antimicrob Agents Chemother. 2017 Aug 24;61(9):e00124-17. doi: 10.1128/AAC.00124-17 (PMC5571324; doi:10.1128/AAC.00124-17)
Supplement: Supplemental material [file AAC.00124-17_zac009176452s1.pdf]

**Supplement Table 1** Effects of Early and Late intervention by PC945 on fungal load (CFU) in lung and galactomannan concentrations in BALF and serum in *Aspergillus fumigatus* infected, immuno-compromised, neutropenic mice

| Treatment Regimen | N | Dose µg/mouse [mg/kg] | Values (% inhibition of response) CFU (mg of lung) | GM in BALF (COI) | GM in serum (COI) |
|---|---|---|---|---|---|
| Vehicle + Conidia | 5 | None | 28.4 ± 16.9 | 4.8 ± 0.40 | 5.3 ± 1.1 |
| Early Intervention | 6 | 2.8 [0.14] | 15.2 ± 13.7 (46) | 0.70 ± 0.39 (85) | 0.81 ± 0.52 (85) |
| | 6 | 14 [0.70] | 2.1 ± 1.6 (93) | 0.37 ± 0.46 (92) | 0.24 ± 0.18 (95) |
| | 6 | 70 [3.5] | 0.8 ± 0.7 (97) | 0.13 ± 0.02 (97) | 0.18 ± 0.07 (97) |
| Late Intervention | 6 | 14 [0.70] | 3.8 ± 1.0 (87) | 0.24 ± 0.06 (95) | 0.29 ± 0.11 (95) |
| | 6 | 70 [3.5] | 1.9 ± 1.7 (93) | 0.22 ± 0.14 (95) | 0.25 ± 0.19 (95) |
| | 6 | 350 [17.5] | 0.5 ± 0.3 (98) | 0.11 ± 0.05 (98) | 0.24 ± 0.11 (95) |

BALF=bronchoalveolar lavage fluid; CFU=colony forming units; COI: cut-off index which was calculated by the formula: Cut-off index=OD in sample / OD in cut-off control provided in kit; GM=galactomannan; N=number.
Data for fungal load and GM are shown as the mean ± standard error (SD); N=5–6.
Percentage inhibition with respect to vehicle (shown in brackets).

**Supplement Table 2** Effects of Early and Late intervention by PC945 on macrophage and neutrophil accumulation into the BALF, IFNγ, IL-17 and MDA levels in the BALF and IL-6 and TNFα levels in the serum of *Aspergillus fumigatus* infected, immuno-compromised, neutropenic mice

| **Treatment Regimen** | **N** | **Dose μg/mouse [mg/kg]** | **Cell numbers in BAL x10$^5$/mL (% inhibition)** | | **Biomarkers in BALF (% Inhibition)** | | | **Biomarkers in serum (% Inhibition)** | |
|---|---|---|---|---|---|---|---|---|---|
| | | | **Macrophage** | **Neutrophil** | **IFNγ (pg/mL)** | **IL-17 (pg/mL)** | **MDA (μg/mL)** | **IL-6 (pg/mL)** | **TNFα (pg/mL)** |
| Vehicle + Conidia | 5 | None | 0.65 ± 0.14 | 0.49 ± 0.09 | 9.2 ± 1.0 | 19.8 ± 3.6 | 1.8 ± 0.2 | 284 ± 112 | 25.6 ± 8.0 |
| Early Intervention | 6 | 2.8 [0.14] | 0.40 ± 0.15 (38) | 0.37 ± 0.04 (24) | 3.7 ± 1.7 (60) | 9.8 ± 5.3 (51) | 0.96 ± 0.32 (47) | 159 ± 73.3 (44) | 11.8 ± 5.9 (54) |
| | 6 | 14 [0.70] | 0.32 ± 0.07 (51) | 0.26 ± 0.12 (47) | 3.0 ± 0.8 (67) | 6.7± 4.9 (66) | 0.57 ± 0.22 (68) | 86.3 ± 46.9 (70) | 7.3 ± 3.5 (71) |
| | 6 | 70 [3.5] | 0.26 ± 0.05 (60) | 0.22 ± 0.04 (55) | 2.5 ± 0.3 (73) | 3.2 ± 0.8 (84) | 0.34 ± 0.05 (81) | 44.5 ± 12.2 (84) | 4.7 ± 0.4 (82) |
| Late Intervention | 6 | 14 [0.70] | 0.43 ± 0.05 (34) | 0.38 ± 0.04 (22) | 4.3 ± 2.2 (53) | 8.5 ± 2.9 (57) | 0.45 ± 0.10 (75) | 51.7 ± 16.8 (82) | 6.2 ± 0.5 (76) |
| | 6 | 70 [3.5] | 0.40 ± 0.11 (38) | 0.34 ± 0.05 (31) | 3.3 ± 0.8 (64) | 4.0 ± 0.8 (80) | 0.37 ± 0.10 (79) | 44.2 ± 11.4 (84) | 5.5 ± 0.7 (79) |
| | 6 | 350 [17.5] | 0.32 ± 0.07 (51) | 0.27 ± 0.08 (45) | 2.1 ± 0.3 (77) | 2.9 ± 0.7 (85) | 0.25 ± 0.05 (86) | 35.9 ± 10.4 (87) | 4.9 ± 0.6 (81) |

BALF=bronchoalveolar lavage fluid; IFNγ=interferon gamma; IL-17=interleukin 17; MDA=malondialdehyde.N=number.
The data are shown as the mean ± standard error (SD); N=5–6. Percentage inhibition with respect to vehicle.

**Supplement Table 3** Effects of Early and/or Late intervention by posaconazole or voriconazole on fungal load (CFU) in lung and galactomannan concentrations in BALF and serum in *Aspergillus fumigatus* infected, immuno-compromised, neutropenic mice

| Treatment Regimen | N | Dose µg/mouse [mg/kg] | Values (% inhibition of response) CFU (mg of lung) | GM in BALF (COI) | GM in serum (COI) |
|---|---|---|---|---|---|
| (Posaconazole) | | | | | |
| Vehicle + Conidia | 5 | None | 21.0 ± 7.3 | 3.4 ± 0.40 | 3.7 ± 0.4 |
| Early Intervention | 6 | 2.8 [0.14] | 13.8 ± 9.7 (34) | 4.4± 0.9 (-29) | 3.0 ± 0.60 (19) |
| | 6 | 14 [0.70] | 9.5 ± 6.3 (55) | 3.5 ± 1.4 (-3) | 2.0 ± 1.3 (46) |
| | 6 | 70 [3.5] | 1.6 ± 0.9 (92) | 0.6 ± 0.3 (82) | 0.3 ± 0.2 (92) |
| (Posaconazole) | | | | | |
| Vehicle + Conidia | 5 | None | 18.7 ± 15.2 | 3.7 ± 1.1 | 3.6 ± 1.1 |
| Late Intervention | 6 | 14 [0.70] | 9.2 ± 12.9 (51) | 2.6 ± 0.5 (30) | 2.7 ± 0.7 (25) |
| | 6 | 70 [3.5] | 1.5 ± 1.0 (83) | 0.6 ± 0.4 (84) | 0.4 ± 0.2 (89) |
| | 6 | 350 [17.5] | 0.4 ± 0.3 (98) | 0.2 ± 0.1 (95) | 0.1 ± 0.0 (97) |
| (Voriconazole) | | | | | |
| Vehicle + Conidia | 5 | None | 23.9 ± 10.5 | 5.9 ± 0.1 | 4.7 ± 0.9 |
| Early Intervention | 6 | 70 [3.5] | 20.6 ± 8.3 (14) | 4.5 ± 1.6 (24) | 2.2 ± 1.3 (53) |
| | 6 | 350 [17.5] | 9.7 ± 1.1 (59) | 3.2 ± 1.3 (46) | 2.3 ± 1.2 (51) |
| | 6 | 1050 [52.5] | 4.9 ± 1.9 (79) | 1.0 ± 0.4 (83) | 0.5 ± 0.2 (89) |

BALF=bronchoalveolar lavage fluid; CFU=colony forming units; COI: cut-off index which was calculated by the formula: Cut-off index=OD in sample / OD in cut-off control provided in kit; GM=galactomannan; N=number.
Data for fungal load and GM are shown as the mean ± standard error (SD); N=5–6.
Percentage inhibition with respect to vehicle (shown in brackets).

# Supplement Figure 1

A

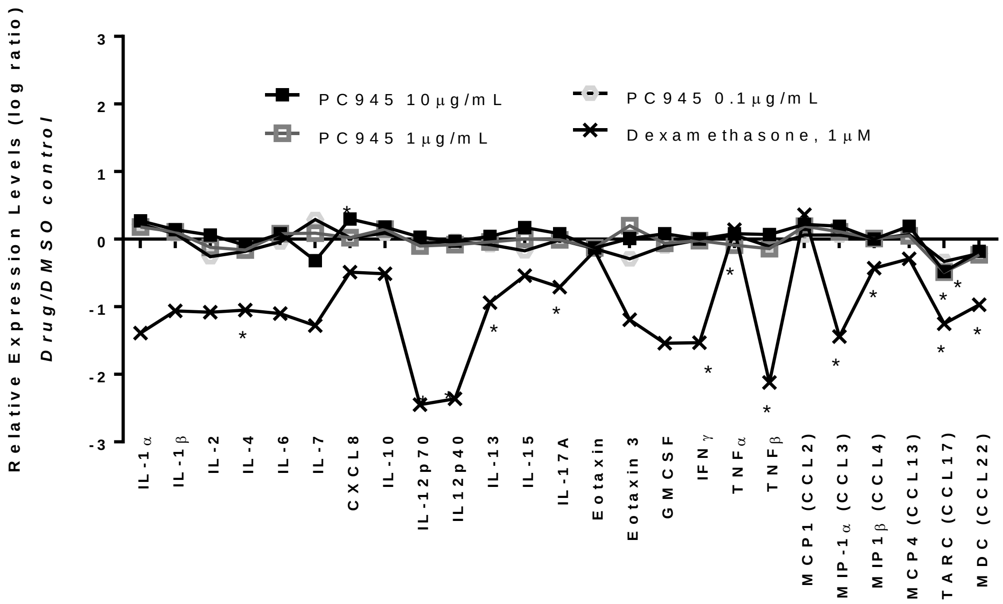


B

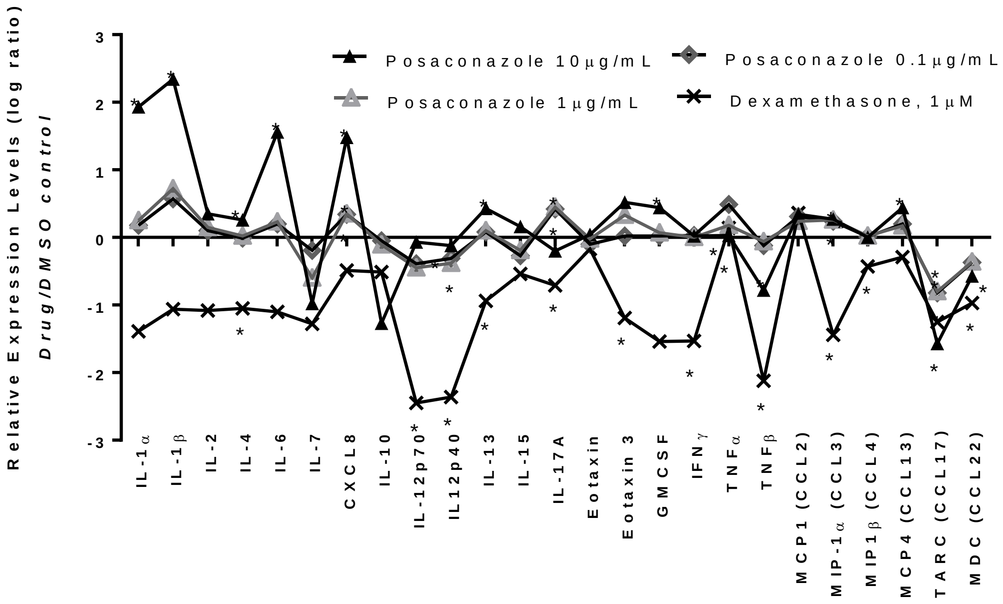


Relative cytokine concentrations in culture supernatant from PBMCs obtained from a healthy subject and stimulated with CytoMix. PC945 or dexamethasone were treated 2hrs before CytoStim stimulation and samples were collected 24hrs post stimulation. Cytokine levels were determined using MSD V-PLEX Plus Human Cytokine 30-Plex Kit and plotted as the value was expressed as log, ratio of the level of cytokines in the presence of drug with that in DMSO control, *p<0.001.
